# Association of circulating hsa-miRNAs with sarcopenia: the SarcoPhAge study

**DOI:** 10.1007/s40520-024-02711-z

**Published:** 2024-03-14

**Authors:** Marjorie Millet, Maxime Auroux, Charlotte Beaudart, Céline Demonceau, Aurélie Ladang, Etienne Cavalier, Jean-Yves Reginster, Olivier Bruyère, Roland Chapurlat, Jean-Charles Rousseau

**Affiliations:** 1 INSERM 1033, Lyon, France; 2 PMO, Lyon, France; 3 Hôpital E. Herriot, Hospices Civils de Lyon, Lyon, France; 4 Clinical Pharmacology and Toxicology Research Unit (URPC), NARILIS, Department of Biomedical Sciences, Faculty of Medicine, University of Namur, Namur, Belgium; 5 WHO Collaborating Center for Epidemiology of Musculoskeletal Health and Aging, Division of Public Health, Epidemiology and Health Economics, University of Liège, Liege, Belgium; 6 Department of Clinical Chemistry, CHU de Liège, University of Liège, Liege, Belgium; 7 University of Lyon, Lyon, France

**Keywords:** Sarcopenia, Circulating hsa-miRNAs, Next generation sequencing, TaqMan qPCR

## Abstract

**Objective:**

To identify a microRNA signature associated to sarcopenia in community-dwelling older adults form the SarcoPhAge cohort.

**Methods:**

In a screening phase by next generation sequencing (NGS), we compared the hsa-miRome expression of 18 subjects with sarcopenia (79.6 ± 6.8 years, 9 men) and 19 healthy subjects without sarcopenia (77.1 ± 6 years, 9 men) at baseline. Thereafter, we have selected eight candidate hsa-miRNAs according to the NGS results and after a critical assessment of previous literature. In a validation phase and by real-time qPCR, we then analyzed the expression levels of these 8 hsa-miRNAs at baseline selecting 92 healthy subjects (74.2 ± 10 years) and 92 subjects with sarcopenia (75.3 ± 6.8 years). For both steps, the groups were matched for age and sex.

**Results:**

In the validation phase, serum has-miRNA-133a-3p and has-miRNA-200a-3p were significantly decreased in the group with sarcopenia vs controls [RQ: relative quantification; median (interquartile range)]: −0.16 (−1.26/+0.90) vs +0.34 (−0.73/+1.33) (*p* < 0.01) and −0.26 (−1.07/+0.68) vs +0.27 (−0.55/+1.10) (*p* < 0.01) respectively. Has-miRNA-744-5p was decreased and has-miRNA-151a-3p was increased in the group with sarcopenia vs controls, but this barely reached significance: +0.16 (−1.34/+0.79) vs +0.44 (−0.31/+1.00) (*p* = 0.050) and  +0.35 (−0.22/+0.90) vs  +0.03 (−0.68/+0.75) (*p* = 0.054).

**Conclusion:**

In subjects with sarcopenia, serum hsa-miRNA-133a-3p and hsa-miRNA-200a-3p expression were downregulated, consistent with their potential targets inhibiting muscle cells proliferation and differentiation.

**Supplementary Information:**

The online version contains supplementary material available at 10.1007/s40520-024-02711-z.

## Introduction

Sarcopenia is a disease defined by progressive decrease in skeletal muscle mass leading to loss of strength and function [1]. Consequently, the individuals with sarcopenia are at increased risk of falls, functional decline, frailty, and mortality [2] Moreover, the amount of metabolically active tissue is reduced by sarcopenia; thus, the risk for metabolic diseases is increased including cardiovascular disease, diabetes, hypertension and hyperlipidemia [3, 4]. The overall prevalence of sarcopenia is estimated between 6 and 22% in adults aged 65 years and increases with age, making sarcopenia a major public health problem [5].

The complex pathogenesis of sarcopenia, however, remains incompletely understood. Moreover, because of several definitions and inadequate screening tools, sarcopenia remains underdiagnosed. Consequently, there is a considerable interest in the identification of specific biologic markers that reflect quantitative and dynamic variations in muscle tissue remodeling [6]. Potential targets include microRNAs (miRNAs). Previous studies have shown that hsa-miRNAs play a pivotal role in the regulation of the major age-associated alterations in muscle pathophysiology including skeletal cell proliferation, differentiation, apoptosis and senescence [2]. These small non-coding RNAs of 22–28 nucleotides in length can silence gene expression by binding to complementary sequence on target messenger RNA transcripts resulting in translational repression or target degradation [7]. One miRNA can inhibit a large number of mRNAs, and one gene can be targeted by multiple miRNAs. Circulating miRNAs are easily accessible and quantifiable with high degree of sensitivity and specificity. Finally, the remarkable miRNA stability in biofluids suggests they could become non-invasive disease biomarkers.

So, the aim of our study was to identify a microRNA signature associated with sarcopenia. For this purpose, we have studied the SarcoPhAge cohort, a prospective longitudinal study following community-dwelling older subjects including 534 participants aged 65 years or older [8], in an initial screening phase and a subsequent validation phase.

## Methods

### Study population

The group for the screening and the validation phases included Belgian participants from the SarcoPhAge study (Sarcopenia and Physical impairments with advancing Age). The main methodology of the SarcoPhAge study has already been described in detail elsewhere [8]. Briefly, the SarcoPhAge cohort is a population-based cohort developed in 2013 in Liège (Belgium). The participants (*n* = 534) were community-dwelling, aged 65 years and older and recruited from press advertisements and general, geriatric, osteoporosis, rehabilitation and rheumatology departments from an outpatient clinic in Liège, Belgium. No specific exclusion criteria related to health or demographic characteristics were applied, except the exclusion of individuals with an amputated limb or with a body mass index (BMI) above 50 kg/m^2^, which was required for X-ray absorptiometry. The study was approved by the Ethics Committee of the Teaching Hospital of the University of Liège (reference 2012/277) with two amendments in 2015 and 2018. All volunteers gave their written informed consent. The participants were followed up annually and a clinical research assistant performed physical examinations and health questionnaires to gather sociodemographic and anamnestic data. The diagnosis of sarcopenia was performed according to the initial definition of 2010 from the European Working Group on Sarcopenia in Older People (EWGSOP) [9]. Muscle mass [Skeletal muscle index measured with daily-calibrated dual energy X-ray absorptiometry (DXA); cut-offs of 7.26 kg/m^2^ for men and 5.5 kg/m^2^ for women] [10], muscle strength (handgrip strength, measured with daily-calibrated JAMAR dynamometer; cut-offs of 30 kg for men and 20 kg for women) [11] and physical performance [short physical performance battery (SPPB); cut-off for the diagnosis of sarcopenia 8 points or less out of a maximum of 12 points] [12] were measured in all the subjects (Table 1).

**Table 1:** Baseline characteristics of individuals in the screening phase (NGS analysis) (A) and the validation phase (qPCR analysis) (B)

(A) Screening	Sarcopenia, *n* = 18	No sarcopenia, *n* = 19	*p* value
Age (years)	79.6 ± 6.8	76.4 ± 6 3	0.14
Sex (women)	9 (50%)	10 (53%)	0.87
SPPB cut-off			
SPPB > 8 points	6 (33.3%)	18 (94.7%)	<0.0001
SPPB ≤ 8 points	12 (66.7%)	1 (5.3%)	
Grip strength maximum (kg)			
Women	20.0 ± 4.4	35.2 ± 11.1	0.007^a^
Men	22.7 ± 4.6	43.6 ± 7.6	<0.0001
Grip strength cut-off			
Grip strength > 20 or 30 kg	0 (0.0%)	18 (94.7%)	<0.0001^b^
Grip strength < 20 or 30 kg	18 (100.0%)	1 (5.3%)	
SMI (kg/m^2^)			
Women	4.9 ± 0.3	7.1 ± 0.7	<0.0001
Men	6.4 ± 0.6	8.7 ± 1.1	0.0002
SMI cut-off			
SMI > 7.26 or 5.5 kg/m^2^	0 (0.0%)	19 (0.0%)	<0.0001^b^
SMI < 7.26 or 5.5 kg/m^2^	18 (100%)	0 (0.0%)	

(B) validation	Sarcopenia, *n* = 92	No sarcopenia, *n* = 92	*p* value
Age (years)	75.8 ± 6.6	75.4 ± 6.2	0.71
Sex (women)	54 (59%)	55 (60%)	0.88
SPPB cut-off			
SPPB > 8 points	39 (42.4%)	72 (78.3%)	< 0.0001
SPPB ≤ 8 points	53 (42.4%)	20 (21.7%)	
Grip strength maximum (kg)			
Women	17.3 ± 3.1	22.1 ± 6.1	< 0.0001^a^
Men	29.0 ± 7.2	38.2 ± 8.9	< 0.0001
Grip strength cut-off			
Grip strength > 20 or 30 kg	14 (15.2%)	66 (71.7%)	<0.0001
Grip strength < 20 or 30 kg	78 (84.8%)	26 (28.3%)	
SMI kg/m^2^			
Women	5.1 ± 0.3	6.4 ± 0.9	<0.0001^a^
Men	6.6 ± 0.5	8.0 ± 0.9	<0.0001
SMI cut-off			
SMI > 7.26 or 5.5 kg/m^2^	0 (0.0%)	80 (87.0%)	< 0.0001
SMI < 7.26 or 5.5 kg/m^0^	92 (100%)	12 (13.0%)	

^a^Mann–Withney *U* test performed due to the non-normality

^b^Fisher’s exact test performed because *N* < 5

SPPB: For the physical performance, we used the short physical performance battery (SPPB) test. This test is composed of three separate tests: balance, 4-m gait speed and chair stand tests. A score between 0 and 4 is assigned for each test, and the 3 tests are weighted equally. As suggested, the cut-off for the diagnosis of sarcopenia for this test, is a score of 8 points or less out of a maximum of 12 points [12]. Gait speed is also proposed by the EWGSOP [9] as a tool to assess physical performance. Given the fact that gait speed is one of the components of the SPPB test, we chose to use the SPPB test as diagnostic tool

Grip strength: as recommended by the EWGSOP [9], we measured subjects’ handgrip strength to determine their muscle strength. Therefore, we used a hydraulic dynamometer (acquired from Saehan Corporation, MSD Europe Bvba, Belgium), calibrated at the beginning of the study for 10, 40 and 90 kg, that subjects had to squeeze as hard as possible three times with each hand (dominant and nondominant). We used the highest result out of the six measurements recorded in our analysis [11]. For the diagnosis of sarcopenia, we used the recommended cut-offs of 30 kg for men and 20 kg for women

SMI: Skeletal muscle index calculated by dividing the appendicular skeletal muscle mass (ASM) by the height squared. The proposed cut-off of 7.26 kg/m^2^ for men and 5.5 kg/m^2^ for women were used [10]

### The hsa-miRNA analysis by next generation sequencing (NGS) : the screening phase

#### Selection of patients

**Fig. 1:**
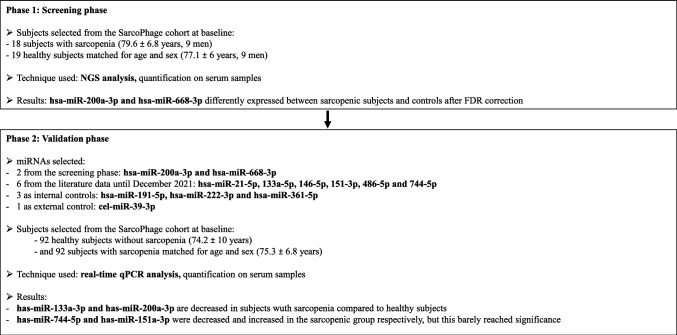
Flow diagram describing the methodology

The expression levels of hsa-miRNAs in serum were measured in a subset of participants, 19 healthy subjects without sarcopenia and 18 subjects with sarcopenia. The number of samples used in this screening phase is comparable to studies previously carried out in our laboratory [13–15] and by other authors [16–21]. Moreover, we have selected a representative sub-group of our cohort in order to detect by NGS the miRs with expression significantly different between controls and sarcopenic subjects. Consequently, we do not know which miRs will be of interest, nor the magnitude of its expression between the two groups. As a result, we cannot calculate a sample size. Both groups were matched for age and sex without any other selection criteria. The flow chart for the design of the experimental study is reported in Fig. 1.

#### NGS analysis

Total RNA was extracted from 400 µl of serum with the hsa-miRCURY Biofluids extraction kit (Exiqon®, Danemark) and analyzed by small RNA sequencing on a NextSeq500 sequencing instrument (Illumina platform). After RNA conversion into hsa-miRNA NGS libraries using NEBNEXT library generation kit, cDNA was pre-amplified prior to library purification and quantification (for detailed procedures of extraction, library preparation and purification, normalization and quality controls, see supplementary data 1). Measurements were expressed as tags per million (TPM) in which the number of reads for a particular hsa-miRNA is divided by the total number of mapped reads in a sample and multiplied by 10^6^. The miRNA level was further normalized by the trimmed mean of *m* values (TMM) method and compared between groups by the TMM ratio expressed as relative data as Log_2_ (Fold change) (LogFC).

### The hsa-miRNA analysis by real-time quantitative polymerase chain reaction: the validation phase

#### Selection of patients

Expression levels of serum hsa-miRNA were measured in 92 subjects with sarcopenia corresponding to the total number of individuals with sarcopenia in the SarcoPhAge cohort and in 92 healthy subjects matched for age and sex.

#### Preselection of hsa-miRNAs

In the validation phase, we have planned to validate the has-miRNAs significantly differentially expressed in the screening phase between patients with sarcopenia and controls. In this screening phase, the prespecified threshold of significance *p* < 0.05 was used after false discovery rate correction using the Benjamini–Hochberg method. Then, we selected 6 additional hsa-miRNA by critical assessment of literature to highlight hsa-miRNAs that are differentially expressed in biofluids of patients with sarcopenia compared with controls [2, 22–28] respectively: 21-5p, 133a-5p, 146a-5p, 151a-3p, 486-5p and 744-5p (for miRNA sequences, see supplementary Table 1).

### Hsa-miRNAs quantification by real-time quantitative polymerase chain reaction (RT-qPCR)

Total RNA was extracted from 100 μl serum with the Nucleospin miRNA plasma kit (Macherey–Nagel®) according to manufacturer’s protocol. A lysis buffer solution containing 1 μl of glycogen as RNA carrier and a synthetic spike-in control RNA (cel-miRNA-39-3p, 0.1 ng) as exogenous control was added to serum. After protein precipitation and washes, RNA was eluted from the microcolumn and stored at −80 °C. The cDNA was synthesized from serum total RNA using a TaqMan Advanced miRNA cDNA synthesis kit (Applied Biosystems, ThermoFisher Scientific). The qPCR amplification was performed on cDNA obtained by miRNA-Amp reaction of reverse transcription reaction, using Fast Advanced Master Mix and TaqMan Advanced miRNA Assays (Applied Biosystems, ThermoFisher Scientific). Hsa-miRNAs quantification was performed by the TaqMan® Advanced miRNA technology (Applied Biosystems, ThermoFisher Scientific) in duplicate of 8 hsa-miRNAs target, 3 hsa-miRNAs endogeneous controles and 1 miRNA as exogeneous control by real-time PCR reaction on a Quantstudio 7 Flex (Applied Biosystems) according to the manufacturer’s protocol. We used the software Expression Suite (Applied Biosystems) to express the hsa-miRNA level as relative quantification (RQ). The threshold cycle (Ct) value of each hsa-miRNA were normalized with the mean of expression level of three endogenous controls. RQ was calculated as 2^–ΔΔCT^, with ΔCT = (CT hsa-miRNA – CT mean of endogenous controls) and ΔΔCT = (ΔCT of the hsa-miRNA –ΔCT mean of the hsa-miRNAs through all samples) and converted as FC = Log2(2^–ΔΔCT^). Data were normalized with the mean of expression level of three endogenous hsa-miRNAs: hsa-miR-191-5p, hsa-miR-222-3p and hsa-miR-361-5p, that are known to be ubiquitously expressed and without reported impact on sarcopenia disease or bone [13, 14]. The exogenous spike cel-miRNA-39-3p was used as a qPCR quality control (for protocol details, see supplementary data 1 and for data normalization, see supplementary Fig.1).

### Statistical analysis

#### NGS (screening phase) and qPCR (validation phase) analysis

In the NGS step, analysis of hsa-miRNAs was performed by Qiagen using the EdgeR software package. For RT-qPCR, RQ was calculated with Expression-Suite Software. A hsa-miRNA with a *p* value ≤ 0.05 and a false discovery rate of 5% (Benjamini–Hochberg FDR correction for NGS approach) was considered as differentially expressed. Wilcoxon tests were used to compare hsa-miRNA levels between subjects with and without sarcopenia because of the skewness of the data.

#### Baseline characteristics of subjects included in this study

Normality of the variables was assessed using a Kolmogorov–Smirnov test, observation of the difference between the mean and the median, histogram and QQ plot. Qualitative variables are presented as numbers (*N*) and percentages (%) and continuous variables are expressed as medians and quartiles. We compared the characteristics between healthy subjects and individuals with sarcopenia using the Mann–Withney *U* test for quantitative variables. Statistical significance was defined as a two-tailed *p* value < 0.05.

The analyses, graphics and figures were performed using GraphPad Prism version 8.4.3 for Windows, GraphPad Software, San Diego, California, USA, http://www.graphpad.com.

## Results

Baseline characteristics of participants are displayed in Table 1. There was no sizeable difference between these age- and sex-matched individuals.

### Screening: serum hsa-miRNA profiling of subjects with sarcopenia and control individuals

Expression levels of serum hsa-miRNA were measured in 19 healthy subjects without sarcopenia (77.1 ± 6 years, 9 men) and in 18 subjects with sarcopenia (79.6 ± 6.8 years, 9 men). We identified 383 has-miRs with an expression level ≥ 1 TPM and 196 with an expression level ≥ 10 TPM in all samples. When we compared both groups, 43 hsa-miRNAs showed differential expression (*p* < 0.05) between controls and individuals with sarcopenia, 31 up-regulated and 12 down-regulated in the group with sarcopenia vs controls. After the Benjamini–Hochberg FDR correction, hsa-miRNA-200a-3p and hsa-miRNA-668-3p remained significantly downregulated in the group with sarcopenia compared to the control group (*p *<0.05, FDR at 5%) (Table 2).

**Table 2:** Differential expression of the miR levels in the serum from healthy subjects and subjects with sarcopenia analyzed by next generation sequencing

miRNAs	Control (TMM)	Sarcopenic (TMM)	logFC	*p* value	FDR
**hsa-miR-668-3p**	**3.820**	**0.230**	**3.287**	**1.720 E−06**	**1.018 E−03**
**hsa-miR-200a-3p**	**146.330**	**50.500**	**1.535**	**5.739E−05**	**1.699 E−02**
hsa-miR-6833-3p	0.250	2.850	−3.122	4.772E−04	9.416 E−02
hsa-miR-1246	135.040	293.180	−1.116	9.589 E−04	1.419 E−01
hsa-miR-4638-5p	0.370	3.450	−3.053	1.250 E−03	1.480 E−01
hsa-miR-509–3-5p	0.100	2.330	−3.702	1.568 E−03	1.547 E−01
hsa-miR-6821-3p	0.040	1.380	−3.593	1.935 E−03	1.637 E−01
hsa-miR-4433-3p	4.040	11.630	−1.514	2.608 E−03	1.930 E−01
hsa-miR-885-5p	41.430	14.990	1.468	3.516 E−03	2.313 E−01
hsa-miR-150-5p	526.010	1271.250	−1.273	3.965 E−03	2.348 E−01
hsa-miR-3591-5p	1.870	0.380	1.989	8.071 E−03	3.690 E−01
hsa-miR-31-5p	0.140	1.510	−2.841	8.625 E−03	3.690 E−01
hsa-miR-140-3p	1288.650	2243.880	−0.800	8.795 E−03	3.690 E−01
hsa-miR-181b-5p	248.470	377.000	−0.602	8.915 E−03	3.690 E−01
hsa-miR-29b-3p	15.010	38.780	−1.368	9.450 E−03	3.690 E−01
hsa-miR-146b-5p	1362.870	2484.160	−0.866	9.974 E−03	3.690 E−01
hsa-miR-15b-3p	64.730	105.750	−0.707	1.261 E−02	4.350 E−01
hsa-miR-4664-3p	5.360	1.410	1.837	1.460 E−02	4.350 E−01
hsa-miR-1285-3p	13.510	25.050	−0.901	1.477 E−02	4.350 E−01
hsa-miR-181a-5p	3903.140	5312.120	−0.445	1.558 E−02	4.350 E−01
hsa-miR-4669	1.390	8.030	−2.422	1.567 E−02	4.350 E−01
hsa-miR-629-5p	603.340	861.850	−0.515	1.656 E−02	4.350 E−01
hsa-miR-29b-2-5p	3.100	0.750	1.857	1.690 E−02	4.350 E−01
hsa-miR-155-5p	206.090	302.870	−0.555	1.798 E−02	4.435 E−01
hsa-miR-1273 g-3p	4.910	1.630	1.570	1.900 E−02	4.500 E−01
hsa-miR-203a	74.690	219.060	−1.554	2.141 E−02	4.874 E−01
hsa-miR-186-5p	1138.270	1749.830	−0.620	2.374 E−02	5.204 E−01
hsa-miR-142-5p	2176.960	3522.360	−0.694	2.768 E−02	5.852 E−01
hsa-miR-181c-3p	4.700	12.170	−1.345	3.131 E−02	6.202 E−01
hsa-miR-205-5p	22.210	85.530	−1.949	3.143 E−02	6.202 E−01
hsa-miR-1292-5p	26.360	42.390	−0.682	3.579 E−02	6.407 E−01
hsa-miR-101-3p	901.930	1587.000	−0.815	3.662 E−02	6.407 E−01
hsa-miR-543	1318.830	810.030	0.703	3.699 E−02	6.407 E−01
hsa-miR-211-5p	0.340	2.720	−2.737	3.742 E−02	6.407 E−01
hsa-miR-671-5p	1.500	4.220	−1.378	3.868 E−02	6.407 E−01
hsa-miR-29c-3p	10.360	20.190	−0.958	3.896 E−02	6.407 E−01
hsa-miR-107	126.140	191.000	−0.599	4.272 E−02	6.692 E−01
hsa-miR-485-3p	112.840	53.340	1.081	4.497 E−02	6.692 E−01
hsa-miR-485-5p	151.120	95.240	0.668	4.561 E−02	6.692 E−01
hsa-miR-224-5p	140.780	89.380	0.654	4.741 E−02	6.692 E−01
hsa-miR-378a-3p	660.340	927.940	−0.491	4.752 E−02	6.692 E−01
hsa-miR-342-5p	396.460	555.760	−0.487	4.761 E−02	6.692 E−01
hsa-miR-382-5p	216.140	141.400	0.610	4.861E−02	6.692 E−01

When we compared the sarcopenic vs healthy groups, 43 miRs showed differential expression (*p* < 0.05) between healthy subjects and subjects with sarcopenia. After Benjamini–Hochberg false discovery rate (FDR) correction, hsa-miR-200a-3p and hsa-miR-668-3p remained significantly different between healthy subjects and subjects with sarcopenia (*p* < 0.05, FDR at 5%) (in bold)

### Validation: differential expression of candidate hsa-miRNAs in serum of subjects with sarcopenia and control individuals

**Fig. 2:**
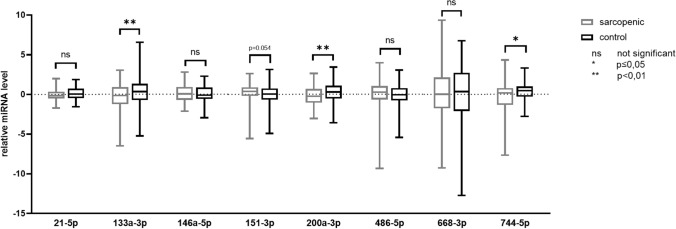
The serum expression levels of the eight miRs analyzed in the validation phase in individual groups of subjects with or without sarcopenia after normalization with the three control miRs and standardized to cel-miRNA-39. Data are shown using a box-and-whisker plot where boxes show the 25th, 50th (median) and 75th percentiles, and the ends of the whiskers indicate the maximum and minimum

Expression levels of serum hsa-miRNA were measured in 92 healthy subjects without sarcopenia (74.2 ± 10 years) and in the 92 subjects with sarcopenia (75.3 ± 6.8 years). Both groups were matched for age (*p* = 0.71) and sex (*p* = 0.88). There was no significant difference between the two groups, so we made no further adjustment. Serum has-miRNA-133a-3p and has-miRNA-200a-3p were significantly decreased in the group with sarcopenia vs controls (RQ: relative quantification; median [Interquartile range]: −0.16 (−1.26/+0.90) vs +0.34 (−0.73/+1.33) (*p *<0.01) and −0.26 (−1.07/+0.68) vs +0.27 (−0.55/+1.10) (*p *<0.01), respectively. Has-miRNA-744-5p was decreased and has-miRNA-151a-3p was increased in the group with sarcopenia compared to the control group, but this barely reached significance: +0.16 (−1.34/+0.79) vs +0.44 (−0.31/+1.00) (*p *= 0.050) and +0.35 (−0.22/+0.90) vs +0.03 (−0.68/+0.75) (*p* = 0.054) respectively (Table 3 and Fig. 2).

**Table 3:** Differential expression of the miR level in the serum from healthy subjects and subjects with sarcopenia analyzed by quantitative real-time PCR

Target	Sarcopenic group				Control group				*p* value
	Median	25% percentile	75% percentile	*n*	Median	25% percentile	75% percentile	*n*	
hsa.miR.21.5p	−0.14	−0.51	0.35	92	0.06	−0.47	0.74	92	0.085
**hsa.miR.133a.3p**	−**0.16**	−**1.26**	**0.90**	**85**	**0.34**	−**0.73**	**1.33**	**90**	**0.009**
hsa.miR.146a.5p	0.05	−0.72	0.93	92	−0.11	−0.61	0.86	92	0.572
**hsa.miR.151a.3p**	**0.35**	−**0.22**	**0.90**	**92**	**0.03**	−**0.68**	**0.75**	**92**	**0.054**
**hsa.miR.200a.3p**	−**0.26**	−**1.07**	**0.68**	**92**	**0.27**	−**0.55**	**1.10**	**92**	**0.009**
hsa.miR.486.5p	0.27	−0.67	1.05	92	−0.07	−0.79	0.80	92	0.108
hsa.miR.668.3p	0.01	−1.77	2.14	82	0.34	−2.11	2.73	82	0.710
**hsa.miR.744.5p**	**0.16**	−**1.34**	**0.79**	**90**	**0.44**	−**0.31**	**1.00**	**90**	**0.050**

The significant miRs are in bold

## Discussion

We have evaluated by real-time qPCR whether the two differentially expressed hsa-miRNAs (200a-3p and 668-3p) in subjects with sarcopenia observed in the screening step were also aberrantly expressed in the validation cohort. In addition, we measured the expression levels of 6 additional hsa-miRNAs (21-5p, 133a-5p, 146a-5p, 151a-3p, 486-5p and 744-5p) previously highlighted in prior studies as having a dysregulated expression in subjects suffering from sarcopenia.

### Screening phase

The NGS approach revealed that 43 hsa-miRNAs had serum levels of expression significantly different between the group with sarcopenia and the healthy subjects. After FDR correction, only 2 of them remained significant downregulated, hsa-miRNA-200a-3p and 668-3p. Of the remaining 41 hsa-miRNAs, twelve were downregulated and twenty-nine upregulated in the group with sarcopenia compared to the healthy group (see Table 2). Most of them are known to be associated with multiple pathways of muscle metabolism.

A first group may be linked to vascular biology dysregulation. Atherosclerosis may be influenced by hsa-miRNAs-155-5p [29], hsa-miRNA-150-5p [30], hsa-miRNA-382-5p [31], hsa-miRNA-205-5p [32, 33] and hsa-miRNA-211-5p [34]. Hsa-miRNA 146-5p [35] and hsa-miRNA-485-3p may affect vascular calcification [36]. Migration, proliferation and differentiation of smooth muscle cells for hsa-miRNA 101-3p, 342-5p and 1246 [37–39] and diseases related to airway smooth muscle cells, asthma and chronic obstructive pulmonary disease may relate to hsa-miRNA 101-3p, 140-3p, 155-3p, 224-5p and 509-5p [40–44].

The second group of hsa-miRNAs included those involved in heart disease as a cardioprotective mechanism for hsa-miRNA 342-5p [45], anti-cardiomyocyte apoptosis after infarction for hsa-miRNA 140-3p [46], inhibition of collagen formation after a myocardial injury for hsa-miRNA 142-5p [47] and hsa-miRNA 485-5p [48], atrial fibrillation for hsa-miRNA 671-5p [49] and chronic heart failure for hsa-miRNA 1285-3p [50].

The third group encompasses hsa-miRNAs related to myoblast metabolism, such as hsa-miRNA 378-3p influencing vascularization in skeletal muscle [51], 885-5p for determining muscle type-specific tissue formation and maintenance [52], hsa-miRNA 203 for muscle differentiation and development [53, 54], hsa-miRNA 205-5p and has-miRNA-342-5p for muscle proliferation and migration of human aortic vascular smooth muscle cells [32, 38] and differentiation of vascular smooth muscle cells regarding hsa-miRNA 142-5p [55].

Finally, the last group includes hsa-miRNAs known to be involved in sarcopenia and exercise as hsa-miRNA 107, hsa-miRNA 29b, hsa-miRNA 181a-5p, hsa-miRNA 15b-3p and hsa-miRNA 186-5p [2]. Moreover, gene ontology (GO) enrichment analysis revealed that bone mineralization, muscle hypertrophy, vascular smooth muscle cell differentiation, regulation of IL-6 synthesis, protein kinase activity and axon guidance are among the main pathways significantly associated with differentially expressed hsa-miRNAs (see supplementary Table 2).

### Validation phase

In the validation step, we analyzed the serum expression levels of 8 hsa-miRNAs at baseline selecting 92 subjects with sarcopenia and 92 healthy subjects matched for age and sex. Among the eight selected hsa-miRNAs, four of them, 21-5p, 146a-5p, 486-5p and 668-3p were not significantly dysregulated between both groups.

In contrast, in the subjects with sarcopenia, the expression of hsa-miRNA-133a-3p and hsa-miRNA-200a-3p were significantly down regulated while the expression of hsa-miRNA-744-5p and hsa-miRNA-151a-3p were down and upregulated respectively but barely significant.

### Potential explanations for the hsa-miRNAs dysregulations in the validation phase

Recently, circulating hsa-miRNAs 146a-5p and 486-5p have been shown to be downregulated in a group of subjects with sarcopenia compared to those of normal and dynapenic groups [22]. The targets of hsa-miRNA-486 suggest that it may modulate the progressive loss of muscle mass in older adults [56, 57]. The hsa-miRNA-146a-5p is an anti-inflammatory hsa-miRNA that negatively regulates the inflammatory response [58]. Moreover, it also modulates cellular senescence and the mitochondrial metabolism [59]. However, we did not find any associations with sarcopenia for hsa-miRNAs 146a-5p and 486-5p in the SarcoPhAge cohort. It could be argued that the subjects did not belong to the same ethnical group, Chinese for Liu et al. and Caucasian in our study. Moreover, the sarcopenia definitions used were different as well as the normalization process for the qPCR quantification.

For the circulating hsa-miRNA-21-5p, four previous studies did not find any association with sarcopenia which is consistent with our findings [22, 60–62]. Comparisons are challenging, however, because the definitions of sarcopenia were not the same, conventional definition for Chen et al. and Asian Working Group for Sarcopenia (AWGS) for the three others and the quantification techniques were different, SYBGreen for He et al. and locked nucleic acid (LNA) probe for Liu et al. in plasma and serum for Chen et al. It could be noticed that hsa-miRNA-21 targets Forkhead box protein O1 (FoxO1) and phosphatase and TENsin homolog (PTEN), two genes involved in sarcopenia dysregulation [63, 64].

Finally, hsa-miRNA-688 has not been quantified in circulation before and we did not manage to quantify it when we measured its level in the largest validation cohort. This may be due to the very low level of its expression in the screening phase, under 4 TPM. However, in silico analysis suggested that hsa-miRNA-668-3p targets nuclear factor-kappa B inhibitor alpha (NFKBIA) that inhibits the transcription factor NF-kB involved in the myogenesis regulation [65]. It also participates in the regulation of skeletal muscle growth by the inhibition of insulin-like growth factor 2 receptor (IGF2R**)** expression [66].

To sum up, this lack of significance for these four hsa-miRNAs can be attributed to differences between our study and the previous ones or we can speculate that the metabolic pathways dysregulated by these hsa-miRNAs do not affect sarcopenia as evaluated in the SarcoPhAge cohort.

However, four hsa-miRNAs had expression significantly different between the two groups. In subjects with sarcopenia, hsa-miRNA-133a-3p and hsa-miRNA-200a-3p expression was downregulated. Both are known to promote myoblast differentiation and proliferation. The hsa-miRNA-133a is primarily produced by skeletal muscle and it belongs to the myomiRNA family. It promotes muscle differentiation and proliferation [28]. In addition, circulating hsa-miRNA-133a was strongly associated with creatine kinase indicating that the muscle damage contributes at least in part to plasma hsa-miRNA-133a levels [25]. As for the hsa-miRNA-200a-3p, it promotes skeletal muscle satellite cells development by targeting transforming growth factor beta 2 (TGF-β2) and regulating the TFG-β2/SMAD signaling pathway [67]. Previously, Liu et al. [22, 68], did not observed a significant dysregulation between healthy subjects and subjects with sarcopenia for has-miRNA-133a in human plasma. Valaskova et al., also looking at hsa-miRNA-133a and b in human plasma [53], found that the severity of muscle performance deterioration was correlated with the decrease of hsa-miRNA-133 expression, similarly to our results.

To sum up, the hsa-miRNA-133a-3p and hsa-miRNA-200a-3p decreases are therefore consistent with their known roles suggesting that these two hsa-miRNAs could be potential circulating markers of sarcopenia.

In contrast, the variations of hsa-miRNA-744-5p and hsa-miRNA-151a-3p in subjects with sarcopenia are the opposite of what we could expect. Hsa-miRNA-744-5p is down regulated while it inhibits myoblast differentiation and myogenesis. The hsa-miRNA-744-5p sequestration by circSNX29 positively upregulates the expression of Wnt family member 5a (Wnt5a) and Ca^2+^/calmodulin-dependent protein kinase II delta (CaMKIIδ) promoting myoblast differentiation [23]. In contrast, Wei et al. showed that hsa-miRNA-151a-3p promotes myoblast proliferation but also it downregulates slow muscle gene expression by targeting ATPase2, an intracellular calcium pump located in the cell sarcoplasmic reticula. This induces the shift in muscle fiber type, upregulating myosin heavy chain beta (MHC-β) stimulating muscle contraction [27].

To sum up, these variations of hsa-miRNA-151a-3p and 744-5p may reflect the adaptation of metabolic activity of muscle cells to lower muscle mass in order to maintain the steady state of muscle quality. These variations are possibly inadequate to compensate for the muscle loss leading to sarcopenia.

Finally, to the best of our knowledge, five studies have been previously performed concerning circulating hsa-miRNAs in serum [60] or plasma [22, 61, 62, 68]. However, the ethnicity of participants, the criteria for sarcopenia evaluation, the qPCR quantification technique and the normalization of the results were different across studies.

### Strengths and limitations of the study

We have investigated the association between the serum hsa-miRNAs expression and sarcopenia in a well-characterized population of subjects, the SarcoPhAge cohort. We used DXA to evaluate muscle mass of subjects. We have chosen to carry out the validation step of our study by analysis of duplicate samples on TaqMan microRNA arrays in order to reduce handling variability to improve the reproducibility between the samples. In addition to the three biologic endogenous controls, we have included a spike in to control for efficiency in the RNA isolation procedure and cDNA synthesis reaction. Moreover, our study combines the validation of NGS results and the first attempt to replicate previously published results, a process that had been lacking in the hsa-miRNA research field. The SarcoPhAge cohort, however, mainly includes volunteers, which can limit the generalizability of our results to all women and men in Belgium. In addition, the loss of muscle mass and poor muscle strength or physical performance in these older adult participants may be only partially attributable to physiologic aging, and the influences of genetic selection, lifestyle, and nutritional status or the differences in other characteristics among the two groups cannot be excluded. Moreover, the current experimental results have not provided direct evidence to clarify how circulating hsa-miRNAs regulate the sarcopenic processes in the older adults.

In conclusion, we have shown a dysregulation of has-miRNA-133a-3p and has-miRNA-200a-3p in older patients with sarcopenia. Our results provide two potential new therapeutic targets, hsa-miRNA-151a-3p and 744-5p for the treatment and/or prevention of sarcopenia.

### Supplementary Information

Below is the link to the electronic supplementary material.

Supplementary file1 (DOCX 384 KB)
