# Supplementary material for: Association of circulating hsa-miRNAs with sarcopenia: the SarcoPhAge study
Source: Aging Clin Exp Res. 2024 Mar 14;36(1):70. doi: 10.1007/s40520-024-02711-z (PMC10940485; doi:10.1007/s40520-024-02711-z)
Supplement: Supplementary file 1 — Supplementary file1 (DOCX 384 KB) [file 40520_2024_2711_MOESM1_ESM.docx]

**Supplementary data.**

**Supplementary Figure 1**


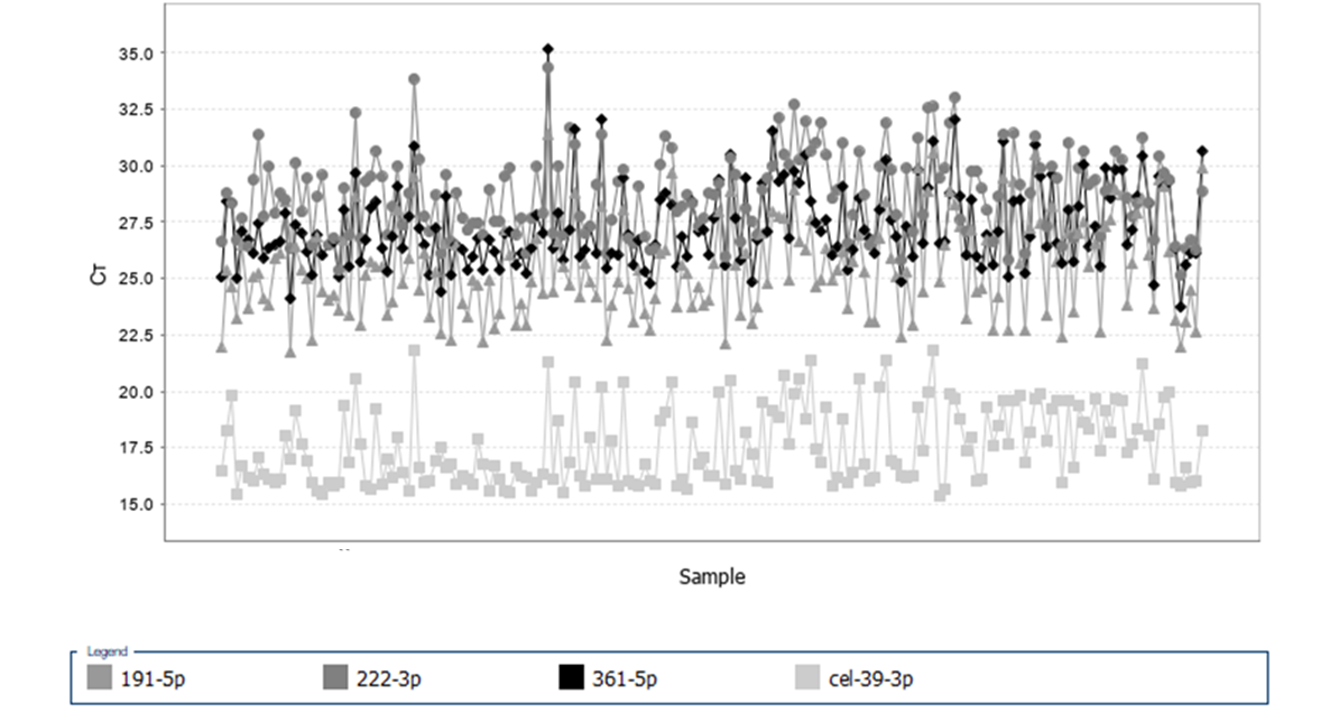


Data were normalized with the mean of expression level of three endogenous hsa-miRNAs: hsa-miR 191-5p, hsa-miR-222-3p and hsa-miR-361-5p that are known to be ubiquitously expressed and without reported impact on sarcopenia disease or bone. The exogenous spike cel-miRNA-39-3p was used as a qPCR quality control. As can be seen, the 3 endogenous controls and the exogenous control vary in the same direction and with the same intensity in each of the sample tested suggesting that the normalization with the mean expression of these 3 miRs is relevant.

**Supplementary Data 1 : Details concerning NGS analysis, RNA extraction and miRNA real-time qPCR analysis.**

**NGS analysis**

According to the manufacturer’s protocol (EXIQON, Denmark), total RNA extraction was performed from 400 µl of serum followed by the miRNA sequencing (Illumina platform). Total RNA (6 μl) extracted from serum with the miRCURY Biofluids extraction kit (Exiqon®, Danemark) was converted into miRNA NGS libraries using NEBNEXT library generation kit (New England Biolabs Inc.) according to the manufacturer’s instructions. Adaptaters containing Unique Molecular Index (UMI) were ligated into 3’-OH and 5’-P prior to the reverse-transcriptase reaction to generate cDNA. The cDNA was pre-amplified with a 18 cycle PCR with primers containing sample specific indexes. The libraries were purified on QiaQuick columns and the insert efficiency evaluated by Bioanalyzer 2100 instrument on high sensitivity DNA chip (Agilent Inc.) The miRNA-cDNA libraries were size fractionated on a LabChip XT (Perkin Elmer) to excise the bands representing adaptors and 15-40 bp insert. The library pool(s) were quantified using the qPCR KAPA Library Quantification Kit (KAPA Biosystems).

The library pool was then sequenced on a Nextseq500 sequencing instrument according to the manufacturer instructions. Raw data was de-multiplexed and FASTQ files for each sample were generated using the bcl2fastq software (Illumina inc.). Measurements were expressed as Tags per million (TPM) in which the number of reads for a particular hsa-miRNA is divided by the total number of mapped reads in a sample and multiplied by 106. The miRNA level was further normalized by the Trimmed Mean of M-values (TMM) method and compared between groups by the TMM ratio expressed as relative data as Log2 (Fold change) (LogFC).

**Validation study: MiRNAs by Real-Time Quantitative Polymerase Chain Reaction**

MiRNAs were quantified on total RNA extracted from serum by TaqMan Advanced miRNA technology (Applied Biosystems, ThermoFisher Scientific). The cDNA was synthesized from 2 μl total RNA for serum, using a TaqMan Advanced miRNA cDNA synthesis kit. Quantification of miRNAs expressed at low copy number was improved by 14 cycles of cDNA amplification in a 2X TaqMan PreAmp master mix containing Megaplex PreAmp primers. The qPCR amplification was performed on 1:20 dilution of cDNA obtained by miR-Amp reaction on 5 μl of the RT reaction, using the 2X Fast Advanced Master Mix and the 20X TaqMan Advanced miRNA Assays. The qPCR amplification was performed on cDNA obtained by miRNA-Amp reaction of reverse transcription reaction, using Fast Advanced Master Mix and TaqMan Advanced miRNA Assays. Hsa-miRNAs quantification was performed by the TaqMan® Advanced miRNA technology (Applied Biosystems, ThermoFisher Scientific) in duplicate of 8 hsa-miRNAs target, 3 hsa-miRNAs endogeneous controles and 1 miRNA as exogeneous control by real time PCR reaction on a Quantstudio 7 Flex (Applied biosystem) according to the manufacturer's protocol (for details see Table 2). The CT (threshold cycle value) was recorded as the cycle number at which the fluorescence generated within a reaction crosses the fluorescence threshold, a fluorescent signal significantly above the ROX™ fluorescence background recorded in each sample. We used the software Expression Suite (Applied Biosystems) to express the miRNA level as relative quantification (RQ). The Ct values of each miRNA were normalized with the mean of expression level of three endogenous controls. RQ was calculated as 2–ΔΔCT, with ΔCT = (CT miRNA – CT mean of endogenous controls) and ΔΔCT = (ΔCT of the miRNA –ΔCT mean of the miRNAs through all samples) and converted as FC = Log2(2–ΔΔCT).

**Supplementary Table 1. Identification of the miRs analyzed in the validation phase and exogenous miR normalizers.**

Each miR is identified by its NCBI accession number and sequence, according to miRBase V20. The pre-designed Taqman Advanced miR Assays used to quantify the serum miR level are specified by their identification number (Applied Biosystems, ThermoFisher Scientific).

| **miR Base ID** | **miRBase Accession Number** | **TaqMan Advanced miRNA Assay (ID)** | **Sequence of the mature miRNA**  **5’—————————3’** |
| --- | --- | --- | --- |
| cel-39-3p | MIMAT0000010 | 478293_mir | UCACCGGGUGUAAAUCAGCUUG |
| hsa-191-5p | MIMAT0000440 | 477952_mir | CAACGGAAUCCCAAAAGCAGCUG |
| hsa-222-3p | MIMAT0000279 | 477982_mir | AGCUACAUCUGGCUACUGGGU |
| hsa-361-5p | MIMAT0000703 | 478056_mir | UUAUCAGAAUCUCCAGGGGUAC |
| hsa-151a-3p | MIMAT0000757 | 477919_mir | CUAGACUGAAGCUCCUUGAGG |
| hsa-21-5p | MIMAT0000076 | 477975_mir | UAGCUUAUCAGACUGAUGUUGA |
| hsa-133a-3p | MIMAT0000427 | 478511_mir | UUUGGUCCCCUUCAACCAGCUG |
| hsa-146a-5p | MIMAT0000449 | 478399_mir | UGAGAACUGAAUUCCAUGGGUU |
| hsa-668-3p | MIMAT0003881 | 479151_mir | UGUCACUCGGCUCGGCCCACUAC |
| hsa-200a-3p | MIMAT0000682 | 478490_mir | UAACACUGUCUGGUAACGAUGU |
| hsa-486-5p | MIMAT0002177 | 478128_mir | UCCUGUACUGAGCUGCCCCGAG |
| hsa-744-5p | MIMAT0004945 | 478200_mir | UGCGGGGCUAGGGCUAACAGCA |

**Supplementary Table 2 : GO analysis of potentially significant terms**

The significant GO terms for the genes targets of miRs found to be differentially expressed between Control and Sarcopenic and their corresponding annotation for Biological process.

| GOID | Term | Pvalue |
| --- | --- | --- |
| [GO:0071236](http://amigo.geneontology.org/amigo/term/GO:0071236) | cellular response to antibiotic | 0.0044 |
| [GO:0030282](http://amigo.geneontology.org/amigo/term/GO:0030282) | bone mineralization | 0.0068 |
| [GO:0030213](http://amigo.geneontology.org/amigo/term/GO:0030213) | hyaluronan biosynthetic process | 0.0105 |
| [GO:1900125](http://amigo.geneontology.org/amigo/term/GO:1900125) | regulation of hyaluronan biosynthetic process | 0.0105 |
| [GO:1900127](http://amigo.geneontology.org/amigo/term/GO:1900127) | positive regulation of hyaluronan biosynthetic process | 0.0105 |
| [GO:0045408](http://amigo.geneontology.org/amigo/term/GO:0045408) | regulation of interleukin-6 biosynthetic process | 0.0114 |
| [GO:0002467](http://amigo.geneontology.org/amigo/term/GO:0002467) | germinal center formation | 0.0116 |
| [GO:0030219](http://amigo.geneontology.org/amigo/term/GO:0030219) | megakaryocyte differentiation | 0.0132 |
| [GO:0044030](http://amigo.geneontology.org/amigo/term/GO:0044030) | regulation of DNA methylation | 0.0137 |
| [GO:0035787](http://amigo.geneontology.org/amigo/term/GO:0035787) | cell migration involved in kidney development | 0.0138 |
| [GO:0090280](http://amigo.geneontology.org/amigo/term/GO:0090280) | positive regulation of calcium ion import | 0.0138 |
| [GO:0042181](http://amigo.geneontology.org/amigo/term/GO:0042181) | ketone biosynthetic process | 0.0155 |
| [GO:0098751](http://amigo.geneontology.org/amigo/term/GO:0098751) | bone cell development | 0.0166 |
| [GO:0061098](http://amigo.geneontology.org/amigo/term/GO:0061098) | positive regulation of protein tyrosine kinase activity | 0.0189 |
| [GO:0002704](http://amigo.geneontology.org/amigo/term/GO:0002704) | negative regulation of leukocyte mediated immunity | 0.0201 |
| [GO:0048842](http://amigo.geneontology.org/amigo/term/GO:0048842) | positive regulation of axon extension involved in axon guidance | 0.0203 |
| [GO:1902669](http://amigo.geneontology.org/amigo/term/GO:1902669) | positive regulation of axon guidance | 0.0203 |
| [GO:0035855](http://amigo.geneontology.org/amigo/term/GO:0035855) | megakaryocyte development | 0.0207 |
| [GO:0035886](http://amigo.geneontology.org/amigo/term/GO:0035886) | vascular smooth muscle cell differentiation | 0.0211 |
| [GO:0016064](http://amigo.geneontology.org/amigo/term/GO:0016064) | immunoglobulin mediated immune response | 0.0216 |
| [GO:0003299](http://amigo.geneontology.org/amigo/term/GO:0003299) | muscle hypertrophy in response to stress | 0.0224 |

The Gene Ontology is a formal representation of species independent knowledge associated with genes and their products. This means the information can be parsed and analyzed by computers to associate the knowledge with the results from biological experiments in order to gain further insight.
